# (+)-Borneol inhibits the generation of reactive oxygen species and neutrophil extracellular traps induced by phorbol-12-myristate-13-acetate

**DOI:** 10.3389/fphar.2022.1023450

**Published:** 2022-11-07

**Authors:** Hanze Chen, Xinxin Xu, Qiwen Tang, Linhui Ni, Shuxia Cao, Yonggang Hao, Li Wang, Xingyue Hu

**Affiliations:** ^1^ Department of Neurology, Sir Run Run Shaw Hospital, School of Medicine, Zhejiang University, Hangzhou, China; ^2^ Department of Neurology, Dushu Lake Hospital Affiliated to Soochow University, Suzhou, China

**Keywords:** neutrophil extracellular traps, borneol, reactive oxygen species, antioxidant, inflammation

## Abstract

**Background and purpose:** Neutrophil extracellular traps (NETs) are special web-like structures that can be generated in both infectious and noninfectious diseases. Previous studies showed that reactive oxygen species (ROS) were crucial in the formation of NETs (NETosis). The purpose of this study is to evaluate the effect of (+)-borneol, an antioxidant, on NETosis.

**Methods:** Human neutrophils were stimulated with phorbol-12-myristate-13-acetate (PMA) to induce NETosis *in vitro*. Neutrophils treated with (+)-borneol at three different time points (−30 min, 0, and 30 min) associated with PMA stimulation were used to examine the effect of (+)-borneol on the formation of NETs. The ROS generation of neutrophils was also measured to explore the potential mechanism of the inhibitory effect of (+)-borneol on NETosis.

**Results:** (+)-Borneol pretreatment inhibited NETosis induced by PMA. Immunofluorescence staining visualized and confirmed the inhibitory effect. (+)-Borneol inhibited the burst of ROS in neutrophils caused by PMA. Suppressing NADPH oxidase or protein kinase C (PKC) eliminated the effect of (+)-borneol on NETosis. Moreover, inhibiting Toll-like receptor 2 (TLR2) led to increased NETosis which can be inhibited by (+)-borneol.

**Conclusion:** (+)-Borneol decreases the ROS level in activated neutrophils and inhibits NETosis triggered by PMA stimulation *in vitro*. (+)-Borneol therapy may be effective in some NET-dependent conditions.

## Introduction

Neutrophils are essential phagocytes that play a crucial role in immune responses. They make up around 60% of white blood cells in normal conditions in humans (Bainton et al., 1971). As the first line of host defense against pathogens, they are activated and recruited to the infectious sites rapidly after pathogen entry (Mayadas et al., 2014). Activated neutrophils can not only phagocytose pathogens into the phagosome for intracellular killing but also release various proteases into the extracellular milieu to enhance their microbicidal function (Ley et al., 2018). In addition, neutrophils can capture and kill microorganisms by releasing special web-like structures called neutrophil extracellular traps (NETs) (Brinkmann et al., 2004). NETs are mainly composed of decondensed chromatin, with granular, cytoplasmic, and nuclear proteins attached (Brinkmann and Zychlinsky, 2007). The formation of NETs, known as NETosis, helps the immune system to eliminate bacteria (Brinkmann et al., 2004; Pilsczek et al., 2010), fungi (Urban et al., 2006), and viruses (Saitoh et al., 2012) more efficiently. Recently, an increasing body of evidence suggests that NETosis can be observed in noninfectious inflammation. In cardiovascular diseases, NETs can act as stimuli and scaffolds for thrombus formation (Fuchs et al., 2010; Döring et al., 2020). In autoimmune diseases, DNA and citrullinated peptides in NETs may be potential sources of autoantibody production (Hakkim et al., 2010; Sur Chowdhury et al., 2014; Grayson and Kaplan, 2016; Gupta and Kaplan, 2016). In cancers, NETs can promote tumor progression and metastasis (Olsson and Cedervall, 2016; Albrengues et al., 2018). Therefore, NETosis is a double-edged sword and should be inhibited in certain circumstances.

There are two main mechanisms of NETosis, vital NETosis and suicidal NETosis. Vital NETosis can be observed in infectious diseases. Neutrophils are still alive and can also crawl and phagocytose pathogens after vital NETosis (Yipp et al., 2012). Suicidal NETosis occurs mainly in noninfectious diseases. Phorbol-12-myristate-13-acetate (PMA) is a good stimulus to induce suicidal NETosis *in vitro*, which has been used originally and widely in studies (Brinkmann et al., 2004; Jorch and Kubes, 2017; Kenny et al., 2017). PMA stimulation initiates the activation of protein kinase C (PKC) and NADPH oxidase (Bianchi et al., 2009; Thiam et al., 2020) which causes reactive oxygen species (ROS) generation. Then, protein arginine deiminase 4 (PAD4), an enzyme that citrullinates histones and decondenses chromatin, is activated (Thiam et al., 2020). Finally, chromatin is released into the cytosol and expelled out of the cell. The purpose of this study is to explore methods to decrease suicidal NETosis.

Borneol, a traditional Chinese medicine, has anti-inflammatory, antioxidative, and analgesic effects (Liu et al., 2021). As one of the borneol products, (+)-borneol is extracted from fresh branches and leaves of *Cinnamomum camphora* (L.) Presl. It is found that (+)-borneol can enhance the activity of antioxidant enzymes such as superoxide dismutase and glutathione peroxidase (Huang et al., 2020). (+)-Borneol can also increase the expression of nuclear factor erythroid 2-related factor 2 (Nrf2) which can activate antioxidant enzymes to alleviate the effects of ROS (Li et al., 2021). Treating primary cultured cortical neurons with (+)-borneol results in decreased ROS generation (Liu et al., 2011). Furthermore, it is noteworthy that ROS are central to PMA-induced NETosis. Stimulating neutrophils with PMA leads to ROS generation (Papayannopoulos, 2018). ROS scavengers can inhibit PMA-induced NETosis (Fuchs et al., 2007; Kenny et al., 2017). Neutrophils isolated from chronic granulomatous disease patients have impaired NADPH oxidase function, and stimulating them with PMA fails to induce NETosis (Bianchi et al., 2009). Taking into consideration the crucial role of ROS in NETosis, we speculate that (+)-borneol reduces NETosis by regulating ROS generation.

In this study, we isolate neutrophils from whole human blood and stimulate them with PMA to generate NETs. Then, we demonstrate the inhibitory effects of (+)-borneol on NETosis, and the underlying mechanism is also explored.

## Materials and methods

### Materials

(+)-Borneol was obtained from the Simcere Pharmaceutical Group. Polymorphprep was obtained from Axis–Shield. RPMI-1640 was obtained from Gibco. PMA and the NETosis assay kit were obtained from Cayman Chemical. HEPES buffer, enhanced cell counting kit-8 (CCK-8), and ROS assay kit were obtained from Beyotime Biotechnology. The Quant-iT PicoGreen dsDNA Assay Kit was obtained from Invitrogen. SYTOX Green nucleic acid stain and Calcein Blue AM were obtained from Maokang Biotechnology. Diphenyleneiodonium chloride (DPI) and Go6976 were obtained from Selleck. C29 and TAK-242 were obtained from MedChemExpress.

(+)-Borneol was dissolved in DMSO (80 mg/ml) and then diluted with RPMI-1640 to different concentrations (1.56, 6.25, 25, 100, and 400 μM). PMA (1 mg/ml in DMSO) was diluted with RPMI-1640 to 100 nM. The vehicle group had no (+)-borneol or inhibitor but the same PMA and DMSO level as the 400-μM (+)-borneol group. In the control group, neutrophils were treated without PMA.

### Donor consent

This study was approved by the Medical Ethical Committee of Sir Run Run Shaw Hospital. Healthy volunteers above the age of 18 were recruited. People were excluded if they have a history of any chronic disorder or take any medication within 2 weeks. Human blood was collected according to the Declaration of Helsinki. Participants involved in the study provided written informed consent before participation.

### Neutrophil isolation

Blood was collected using EDTA-K2 blood collection tubes. Neutrophils were isolated using Polymorphprep, according to the manufacturer’s instructions. Briefly, 5 ml of fresh blood was layered over 5 ml of Polymorphprep. After centrifugation at 600 g for 30 min, the lower band was collected and erythrocytes were removed using RBC lysis buffer (CWBIO). The neutrophils were washed and resuspended in RPMI-1640 supplemented with HEPES buffer.

### Cell viability analysis

Cell viability was evaluated using enhanced cell counting kit-8. The time points were determined according to the instruction. Briefly, 5 × 10^4^/well neutrophils from three healthy volunteers were seeded in 96-well plates and treated with vehicle and different concentrations of (+)-borneol (1.56, 6.25, 25, 100, and 400 μM) for 4 h. A measure of 10 μl/well of CCK-8 solution was added and incubated for 1 h at 37°C. Wells containing no neutrophil but the same volume of RPMI-1640 and CCK-8 solutions were set as a negative control. The absorbance was then measured at 450 nm using a microplate reader (SpectraMax M5, Molecular Device). The results were normalized by dividing the value in the vehicle group and representing as percentages.

### NETosis analysis

NETosis analysis was performed, as previously described (Schulz et al., 2020). Briefly, freshly collected neutrophils were plated in 24-well plates (1 × 10^6^/well). Neutrophils were treated with or without inhibitors such as the NADPH oxidase inhibitor (DPI, 20 μM), PKC inhibitor (Go6976, 1 μM), TLR2 inhibitor (C29, 100 μM), or TLR4 inhibitor (TAK242, 100 μM) for 30 min at 37°C. Then, 400 μM (+)-borneol was added. After incubation for another 30 min, 100 nM PMA was added to each well to stimulate NETosis and incubated for 4 h. At the end of incubation, neutrophils were washed twice with 1 ml RPMI-1640 to remove unbound neutrophil elastase. Then, S7 nuclease (50 U/ml) was added to digest DNA and release NET-associated neutrophil elastase. Following incubation for 30 min, 15 μl EDTA (500 mM) was added to stop the effect of S7 nuclease. The supernatant was collected for neutrophil elastase activity analysis using N-methoxysuccinyl-Ala-Ala-Pro-Val p-nitroanilide. Cell-free DNA (cfDNA) was detected using the Quant-iT PicoGreen dsDNA Assay Kit, according to the instruction. To explore the effect of (+)-borneol, 400 μM of it was added at three different time points (−30 min, 0, and 30 min) associated with PMA stimulation. Relative fold expression of elastase and cfDNA was used in data analysis to make the results comparable.

### Reactive oxygen species assay

Freshly isolated neutrophils were resuspended in RPMI-1640 and plated in 96-well plates (1 × 10^6^/well) for 30 min. Then, neutrophils were treated with 400 μM (+)-borneol for another 30 min, followed by stimulation by 100 nM PMA for 4 h. For ROS assay, 100 μl of DCFH-DA (10 μM, diluted with RPMI-1640) was added to each well. After incubation for 30 min at 37°C, fluorescence was detected at an excitation wavelength of 480 nm and emission wavelength of 520 nm using a microplate reader (SpectraMax M5, Molecular Device).

### Neutrophil extracellular trap imaging

For imaging, 1 × 10^4^ neutrophils were plated in Φ 15-mm glass bottom dishes and incubated in the incubator for 30 min. Then, they were treated with or without 400 μM (+)-borneol for another 30 min. After that, 100 nM PMA was added to induce NETosis. After stimulation for 4 h, neutrophils were washed with 1 ml PBS. We used 5 μM Calcein Blue AM and 0.1 μM SYTOX Green nucleic acid stain to visualize live neutrophils and DNA in NETs, respectively. Images were acquired using a Nikon A1 confocal microscope.

### Statistics

Data from at least three independent experiments are presented as mean ± SEM. GraphPad Prism software (version 8.0) was used for data analysis. Student’s *t*-test and one-way ANOVA were used for two-group and multi-group comparisons, respectively. *p* < 0.05 was considered significant.

## Results

### (+)-Borneol has no effect on neutrophil viability

**FIGURE 1:**
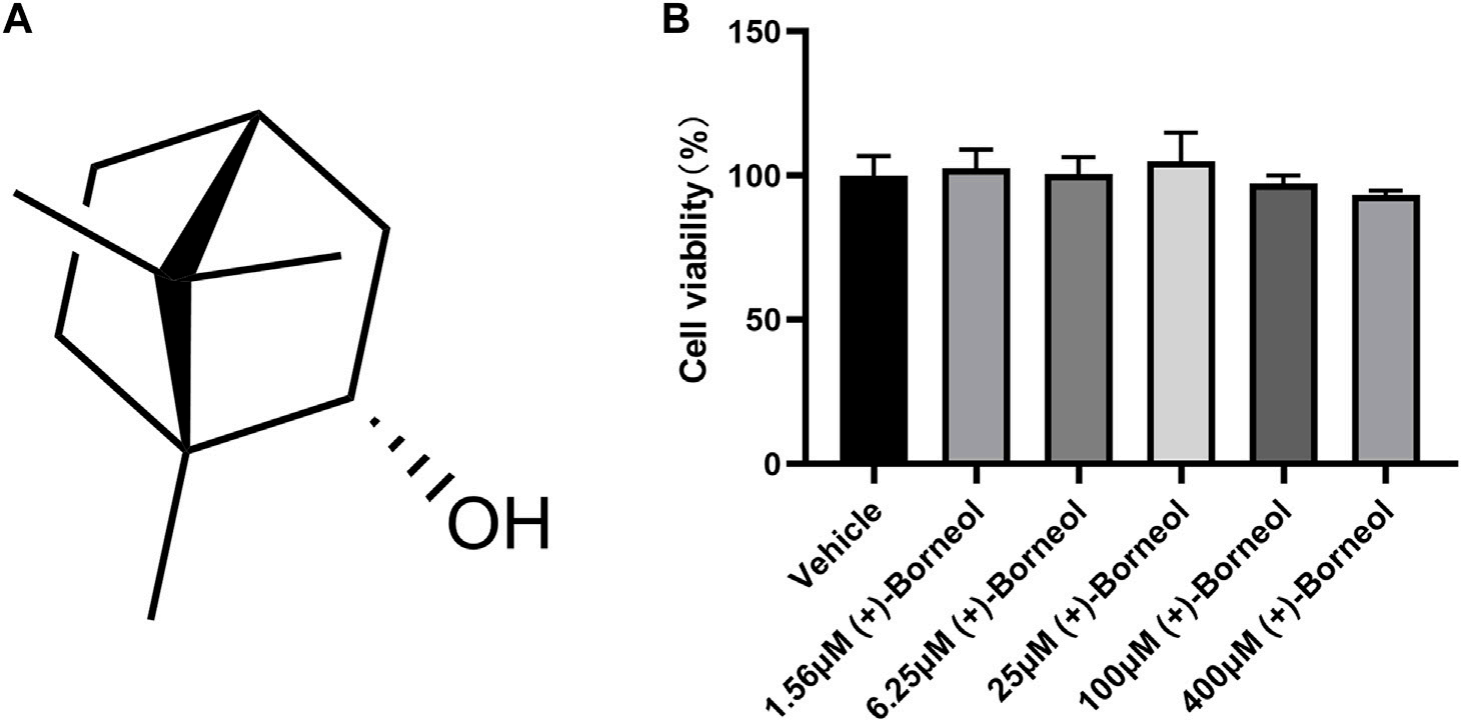
(+)-Borneol has no effect on neutrophil viability. **(A)** Chemical structural formula of (+)-borneol. **(B)** 5 × 104 neutrophils from healthy volunteers were treated with different concentrations of (+)-borneol (1.56, 6.25, 25, 100, and 400 μM) for 4 h, and the cell viability was measured using a CCK-8 assay (*n* = 4).

The chemical structure of (+)-borneol is shown in Figure 1A. To determine the effect of (+)-borneol on neutrophil viability, 5 × 10^4^ neutrophils from healthy volunteers were treated with different concentrations of (+)-borneol (1.56, 6.25, 25, 100, and 400 μM) for 4 h. Then, the viability of neutrophils was measured using CCK-8 assay. As shown in Figure 1B, (+)-borneol did not affect neutrophil viability significantly.

### (+)-Borneol suppresses phorbol-12-myristate-13-acetate-induced NETosis

**FIGURE 2:**
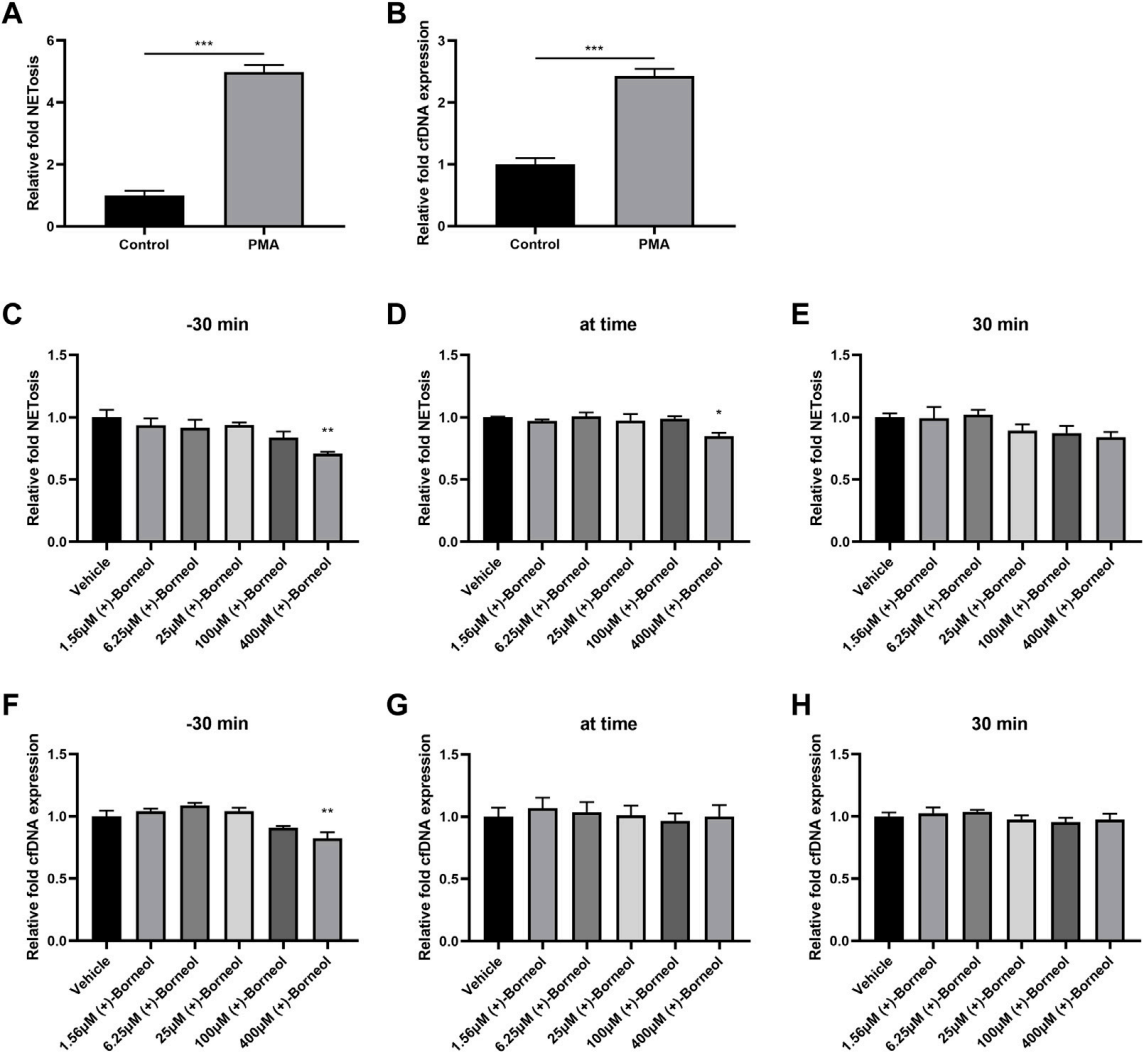
(+)-Borneol reduces PMA-induced NETosis. Neutrophils were stimulated with PMA for 4 h, **(A)** NETosis was measured by the NETosis assay kit, and **(B)** cfDNA was measured by the Quant-iT PicoGreen dsDNA Assay Kit. Neutrophils were treated with (+)-borneol (1.56, 6.25, 25, 100, and 400 μM) at three different time points related to PMA stimulation. NETosis **(C–E)** and cfDNA **(F–H)** were measured, respectively. *p < 0.05, **p < 0.01, and ***p < 0.001 compared with the control or vehicle groups (*n* = 3).

Human neutrophils were isolated and stimulated with 100 nM PMA, an activator of protein kinase C (PKC) and NADPH oxidase. After stimulation for 4 h, the generation of NETs and dsDNA increased significantly compared to the control group (Figures 2A,B). In order to investigate the effect of (+)-borneol on NETosis, different concentrations of (+)-borneol were added to the medium at different time points. In groups where (+)-borneol were added 30 min before PMA stimulation, 400-μM (+)-borneol inhibited NETosis significantly (Figure 2C). Moreover, 400-μM (+)-borneol exhibited similar inhibitory effects in groups where (+)-borneol and PMA were added simultaneously (Figure 2D). However, (+)-borneol had no inhibitory effect when added 30 min after PMA stimulation (Figure 2E). The effect of (+)-borneol on the generation of dsDNA was also evaluated. (+)-Borneol decreased the generation of dsDNA significantly only when added 30 min before PMA stimulation (Figures 2F–H).

### The effect of (+)-borneol on NETosis confirmed by live cell imaging

Immunofluorescence staining was used to visualize and confirm the inhibitory effect of (+)-borneol on NETosis. Neutrophils were pre-incubated with (+)-borneol for 30 min before PMA stimulation. Neutrophils that had no treatment or stimulation were set as control. We visualize live neutrophils and NETs with Calcein Blue AM (cell-permeable) and SYTOX Green nucleic acid stain (cell-impermeable), respectively.

**FIGURE 3:**
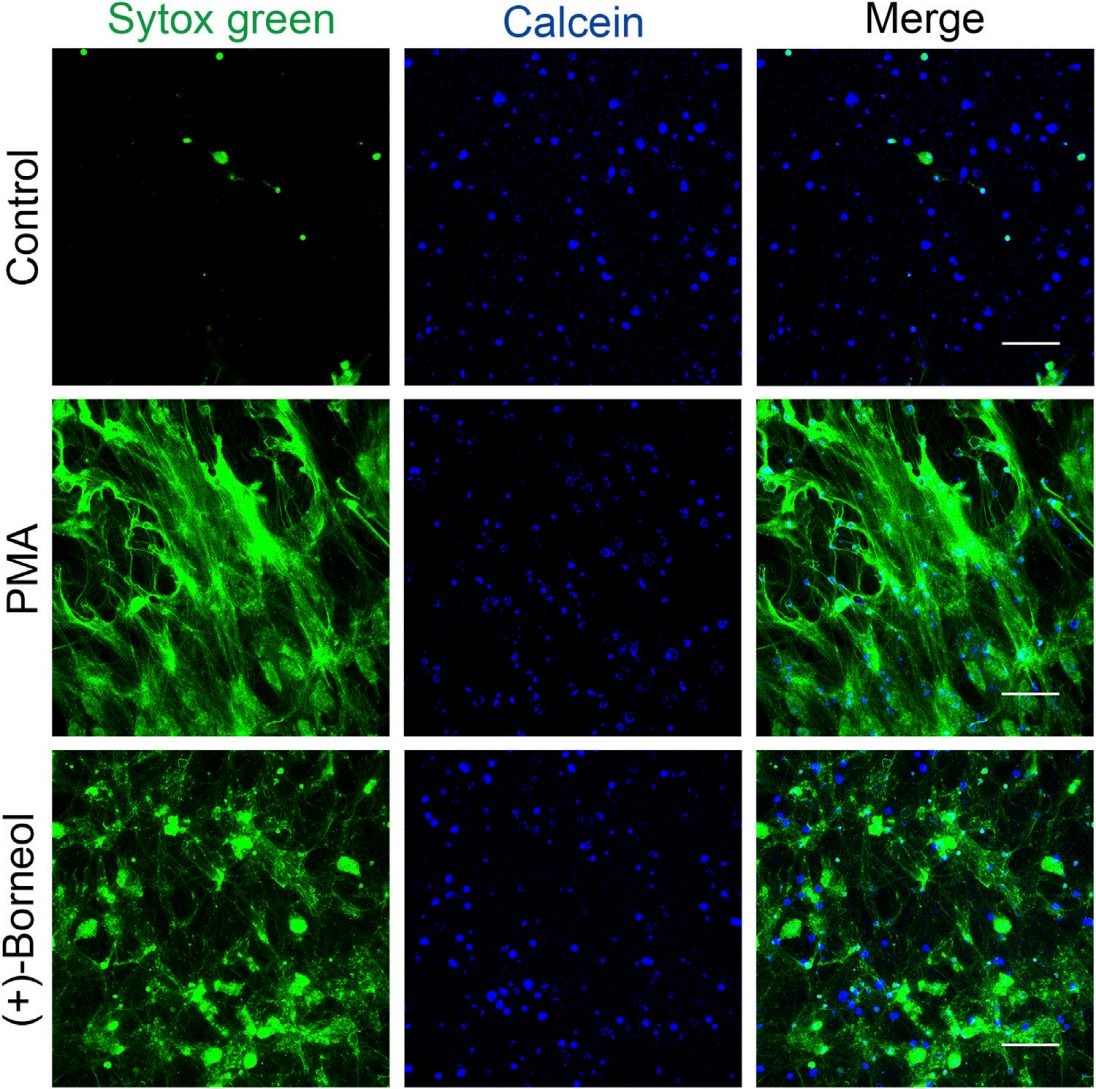
Immunostaining confirms the effect of (+)-borneol on NETosis. Neutrophils that had no treatment or stimulation were set as control. In the PMA group, neutrophils were stimulated with PMA for 4 h. In the (+)-borneol group, neutrophils were preincubated with 400-μM (+)-borneol for 30 min before 100-nM PMA stimulation. We visualized live neutrophils and NETs with Calcein Blue AM (blue) and SYTOX Green nucleic acid stain (green), respectively. Scale bars: 100 μm (*n* = 3).

After PMA stimulation, neutrophils were characterized by cell disruption, chromatin decondensation, and the subsequent massive release of NETs. Consistent with the aforementioned results, more neutrophils with morphological intact survived and NETosis was inhibited when preincubated with 400 μM (+)-borneol (Figure 3).

### (+)-Borneol inhibits the generation of reactive oxygen species

**FIGURE 4:**
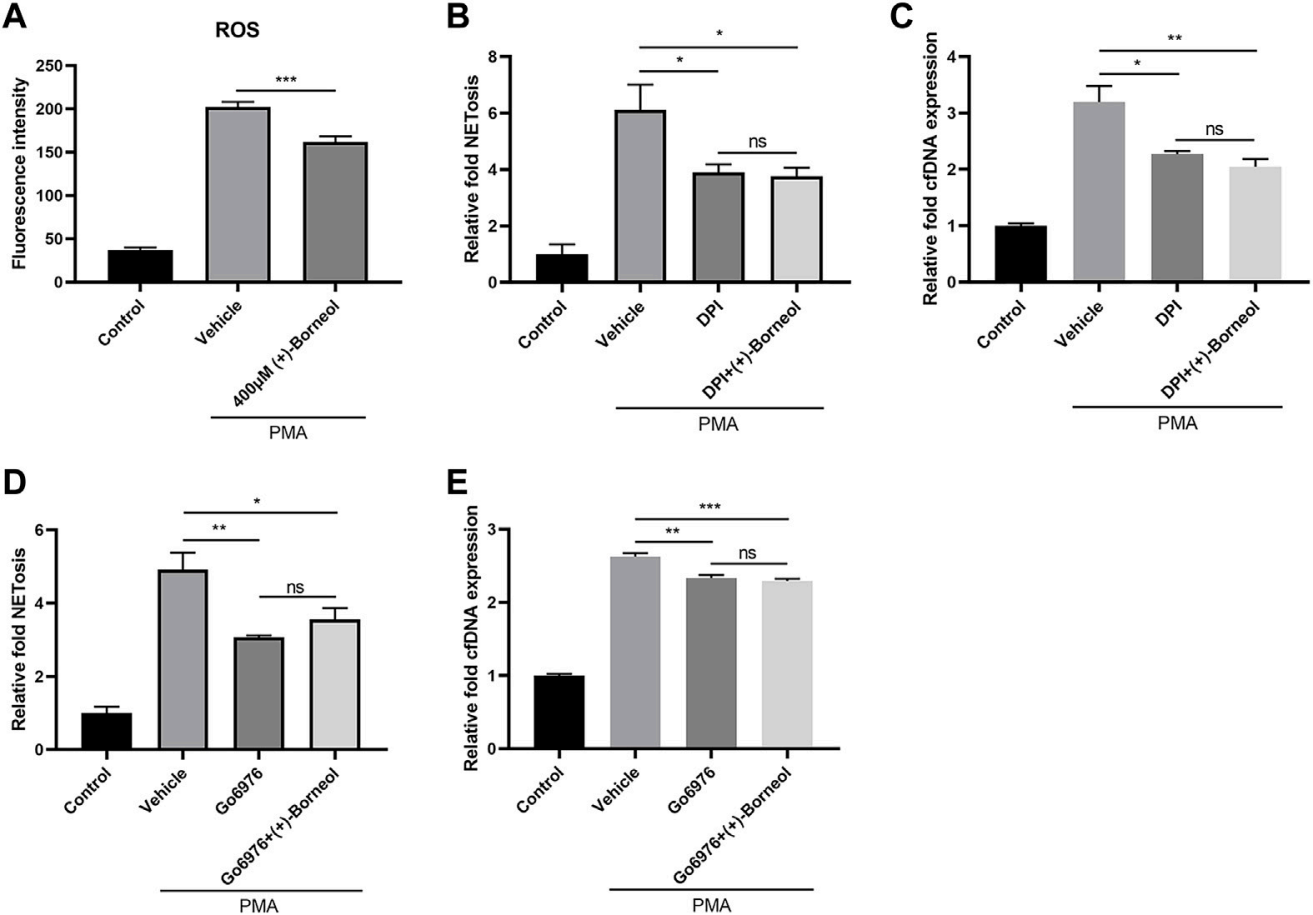
(+)-Borneol inhibits PMA-stimulated ROS generation. **(A)** ROS generation by neutrophils. ROS generation was measured in the presence or absence of (+)-borneol before PMA stimulation. *n* = 4 per group. **(B–E)** NETs and cfDNA produced by neutrophils. *n* = 3 per group. **(B,C)** Neutrophils were preincubated with 20 μM DPI for 30 min, followed by being treated with or without (+)-borneol for another 30 min and then stimulated with PMA. **(D,E)** Neutrophils were pre-incubated with 1 μM Go6976 for 30 min, followed by being treated with or without (+)-borneol for another 30 min and then stimulated with PMA. *p < 0.05, **p < 0.01, and ***p < 0.001; ns = not significant.

Next, we examined the effect of (+)-borneol on the generation of ROS, which plays a crucial role in NETosis. PMA stimulation induced a burst of ROS in neutrophils. However, this PMA-stimulated ROS was inhibited significantly by (+)-borneol (Figure 4A). To explore the effect of (+)-borneol on ROS generation, we treated neutrophils with DPI. Consequently, DPI decreased NETosis induced by PMA, and (+)-borneol cannot enhance the effect of DPI (Figure 4B). Detection of cfDNA showed similar results (Figure 4C). Moreover, PMA-induced NETosis was inhibited by Go6976, a PKC inhibitor. Treating neutrophils with (+)-borneol did not change the inhibitory effect of Go6976 significantly (Figure 4D). Likewise, the cfDNA assay showed consistent results (Figure 4E).

### The effect of (+)-borneol on inhibiting NETosis is independent of Toll-like receptor 2/4

**FIGURE 5:**
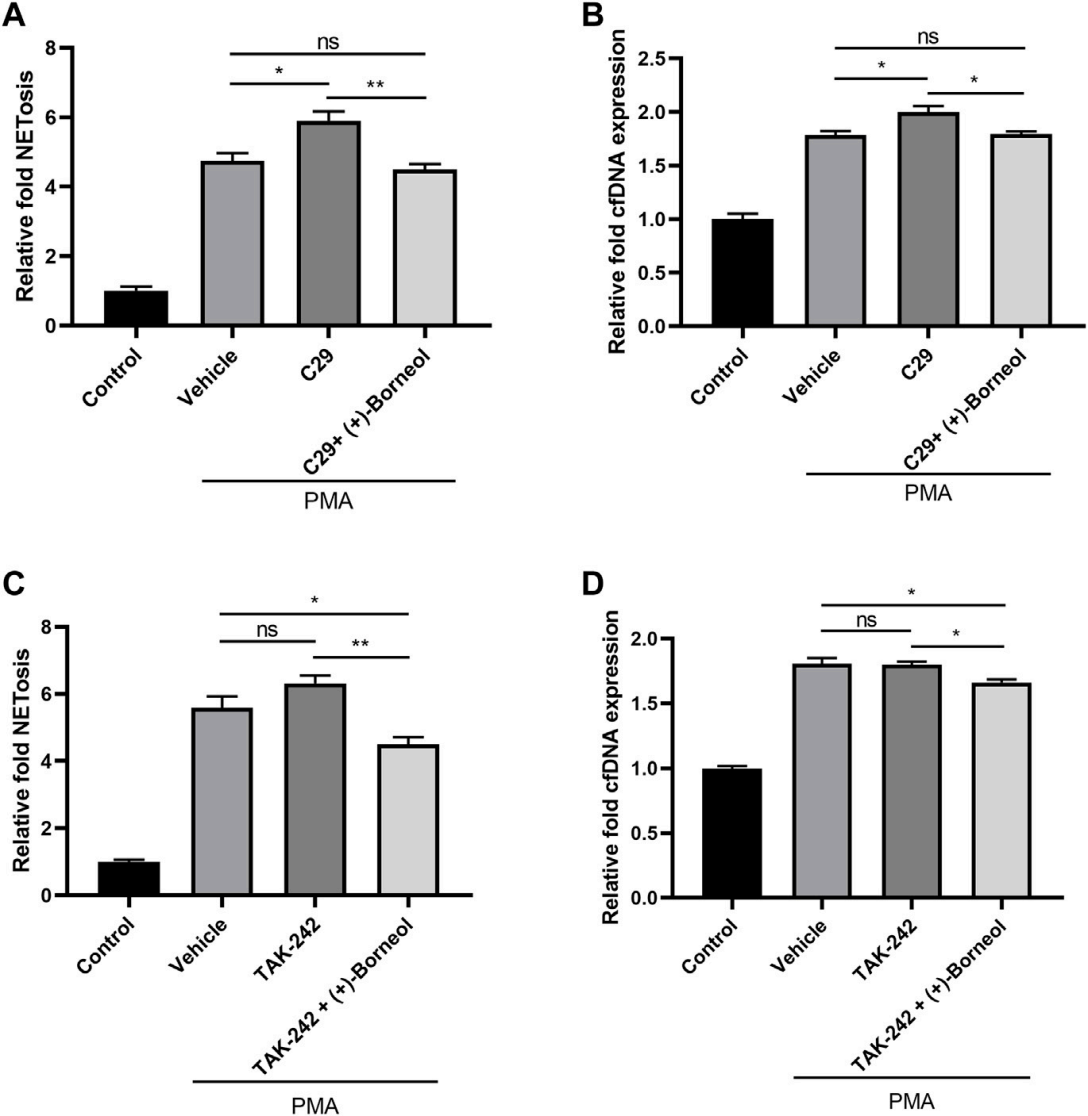
(+)-Borneol suppressed NETosis in a way independent of TLR2/4. **(A,B)** Neutrophils pre-incubated with 100 μM C29 were treated with or without (+)-borneol and then stimulated with PMA. **(A)** NETosis and **(B)** cfDNA were detected after stimulation. **(C,D)** Neutrophils preincubated with 100 μM TAK242 were treated with or without (+)-borneol and then stimulated with PMA. **(C)** NETosis and **(D)** cfDNA were detected after stimulation. *n* = 3 per group; *p < 0.05 and **p < 0.01; ns = not significant.

To explore the role of TLR2/4 on PMA-induced NETosis, we pre-incubated neutrophils with C29 and TAK242 to inhibit TLR2/4. Then, neutrophils were stimulated with PMA for NETosis. It was worth noting that the inhibition of TLR2 increased the generation of NETs and cfDNA induced by PMA compared with PMA treatment alone (Figures 5A,B). However, TAK242 preincubation did not change the level of NETosis and cfDNA (Figures 5C,D). Compared with neutrophils preincubated with C29/TAK242 alone, neutrophils treated with (+)-borneol after preincubation of C29/TAK242 generated fewer NETs and cfDNA after PMA stimulation (Figures 5A–D). These results indicated that (+)-borneol suppressed NETosis in a way independent of TLR2/4.

## Discussion

NETs have been found to play increasingly important roles in many diseases. The effect of NETosis is a double-edged sword that should be taken seriously. In this study, we demonstrated for the first time that (+)-borneol inhibits ROS generation and NETosis of human neutrophils triggered by PMA stimulation *in vitro*.

The stimulus used for NETosis in this study is PMA. PMA stimulation initiates the activation of PKC and NADPH oxidase, which causes ROS generation (Bianchi et al., 2009; Thiam et al., 2020). Afterward, PAD4 is activated by ROS. Meanwhile, neutrophil elastase and myeloperoxidase are released into the cytosol from azurophilic granules. All these responses lead to histone hypercitrullination and chromatin decondensation, followed by NETosis (Jorch and Kubes, 2017). Previous studies have shown that ROS inhibitors block PMA-induced NETosis (Fuchs et al., 2007; Kenny et al., 2017). Neutrophils isolated from chronic granulomatous disease patients have impaired the NADPH oxidase function, and stimulating them with PMA fails to induce NETosis (Bianchi et al., 2009). Therefore, ROS are central to NETosis induced by PMA.

(+)-Borneol is a bicyclic terpenoid that has been shown to synergistically enhance the antitumor and neuroprotective effects of other drugs (Chen et al., 2014; Chen et al., 2015; Lai et al., 2020; Xu et al., 2021). It has been proven to inhibit Aβ-induced ROS generation in SH-SY5Y cells (Hur et al., 2013) and decrease neuronal ROS production caused by oxygen-glucose deprivation (Liu et al., 2011). (+)-Borneol can also increase the expression of Nrf2 (Hur et al., 2013), which can bind to antioxidant response element receptors and induce the expression of antioxidant enzymes to help resist ROS. Moreover, (+)-borneol exerts its antioxidative effect by increasing the superoxide dismutase and glutathione peroxidase activity (Yu et al., 2016). PMA can induce mitochondrial ROS production in neutrophils despite a slight effect on NETosis (Lood et al., 2016). A recent study shows that (+)-borneol reduces mitochondrial ROS generation in endothelial cells (Li et al., 2022).

Taking into consideration the important role of ROS in NETosis and the antioxidative effect of (+)-borneol, we evaluated the effect of (+)-borneol on NETosis. As shown previously, 400-μM (+)-borneol inhibited PMA-induced NETosis without affecting the neutrophil viability. We measured ROS generation and found that (+)-borneol can decrease PMA-stimulated ROS levels in neutrophils. DPI and Go6976 can inhibit NADPH oxidase and PKC, respectively. Treating neutrophils with them can result in a decrease in PMA-induced ROS generation and inhibition of NETosis (Fuchs et al., 2007; Hakkim et al., 2011; Kenny et al., 2017). In this study, we used DPI and Go6976 to inhibit ROS to explore the role of ROS in (+)-borneol’s effect on NETosis inhibition. As shown in Figure 4, the effects of (+)-borneol on NETosis were eliminated in neutrophils pre-treated with DPI or Go6976, which further confirmed the finding mentioned previously. However, it is noteworthy that the inhibitory effect of (+)-borneol on NETosis is time-dependent. A stronger effect was observed when (+)-borneol was added 30 min before PMA stimulation. Thus, (+)-borneol therapy targeting NETosis may be effective in some diseases, especially those with relatively slow neutrophil activation. In future experiments, in order to realize the effect of (+)-borneol *in vivo*, it should be administered before neutrophil activation.

Currently, DNase is the most frequently used inhibitor of NETs (Jorch and Kubes, 2017). It can eliminate DNA, which is the framework of NETs, and the proteins attached in NETs are then released. Thus, the detrimental effect of these proteins cannot be neutralized. As shown in our study, (+)-borneol inhibits ROS generation and NETosis of neutrophils triggered by PMA stimulation. However, the effect of (+)-borneol on NETosis *in vivo* and the specific mechanism await further study.

TLRs located on the cell surface and in endosomes are the first pattern-recognition receptors to be identified (Baral et al., 2014). They detect numerous damage-associated molecular patterns and mediate innate immune responses. TLR2 and TLR4 are two vital members of the neutrophil TLRs (Prince et al., 2011). NETosis can be induced by bacteria, parasites, or lipopolysaccharides (infectious stimuli) *via* TLR2 and/or TLR4 in a few minutes (Jorch and Kubes, 2017). However, the roles TLR2/4 played in the process of PMA-induced NETosis are not clear. In this study, we show that inhibiting TLR4 has no effect on PMA-induced NETosis, which is in line with the previous study (Khan et al., 2017). However, it is interesting that inhibiting TLR2 results in an elevated level of NETosis induced by PMA. Hitherto, TLR2 has not engaged much attention in the process of PMA-induced NETosis. This study indicates that the role of TLR2 in NETosis may be mysterious, and future studies should pay more attention to it because we have not found a reasonable explanation.

There are some limitations to our study. First, we showed that (+)-borneol can inhibit ROS generation of neutrophils, but the exact target of (+)-borneol in neutrophils during NETosis is still unclear. Second, NETs can be induced by many stimuli; we only stimulated neutrophils with PMA. Other stimuli such as lipopolysaccharide (LPS), A23187, and the immune complex can also induce NETosis, and the mechanisms involved are also different. The effect of (+)-borneol on NETosis induced by other stimuli should be studied in the future. However, PMA is a robust NET inducer that has been used originally and widely in studies about NETosis (Brinkmann et al., 2004; Jorch and Kubes, 2017; Kenny et al., 2017). PMA, (auto) antibodies and cholesterol crystals elicit similar pathways in NETosis (Jorch and Kubes, 2017). PMA stimulation can induce suicidal NETosis *in vitro* which can be found in many noninfectious diseases. The purpose of our study is to explore the effect of (+)-borneol on suicidal NETosis. Thus, we selected PMA as the stimulant. Third, according to the Chinese Pharmacopoeia, there are three borneol products: (+)-borneol, (−)-borneol, and (±) borneol. The functions of these products are similar but not identical. However, only the function of (+)-borneol was evaluated in this study. Therefore, our results may not be applicable to other borneol products.

## Conclusion

In summary, our study shows for the first time that (+)-borneol inhibits ROS generation and NETosis of neutrophils triggered by PMA stimulation *in vitro*. This finding indicates that (+)-borneol therapy targeting NETosis may be effective in some diseases.

## Data Availability

The original contributions presented in the study are included in the article/Supplementary Material; further inquiries can be directed to the corresponding authors.
